# Advancing cardiovascular care—key insights from the Netherlands Heart Journal 2025

**DOI:** 10.1007/s12471-025-02012-8

**Published:** 2026-01-07

**Authors:** Pim van der Harst, Clara E. E. van Ofwegen-Hanekamp, Maryam Kavousi, Martin E. W. Hemels, Joris R. de Groot, Peter Damman

**Affiliations:** 1 Department of Cardiology, University Medical Centre Groningen, University of Groningen, Groningen, The Netherlands; 2 Department of Cardiology, Diakonessenhuis, Utrecht, The Netherlands; 3 Department of Epidemiology, Erasmus MC, University Medical Centre Rotterdam, Rotterdam, The Netherlands; 4 Rijnstate Hospital, Arnhem, The Netherlands; 5 Department of Cardiology, Heart Centre, University Medical Centres/University of Amsterdam, Amsterdam, The Netherlands; 6 Department of Cardiology, Radboud University Medical Centre, Nijmegen, The Netherlands; 7 Radboud University Medical Center, Nijmegen, The Netherlands

​
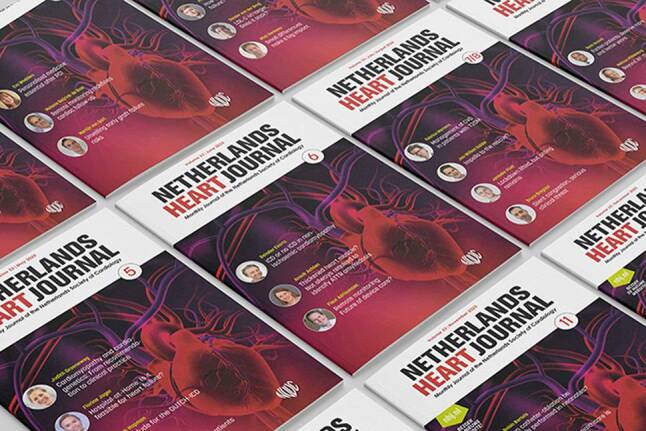


## Introduction

In 2025, the *Netherlands Heart Journal* offered a broad window into how cardiovascular medicine is changing in the Netherlands and beyond. The papers published over the past year show a field that is increasingly data-driven and collaborative, but also more focused on differences between women and men; patients who prefer to be monitored at home rather than in hospital; and families dealing with inherited cardiomyopathies or advanced heart failure.

Several recurring threads run through this year’s volume. One is the growing awareness that sex and gender are not simply statistical modifiers but are central determinants of cardiovascular risk, diagnosis, and treatment response. Another is the steady integration of digital technologies and artificial intelligence into everyday practice, from telemonitoring and hospital-at-home care to AI-enabled imaging and stethoscopes. At the same time, large-scale registries, national consortia and guideline endorsements continue to play a crucial role in understanding real-world outcomes and translating European recommendations into the Dutch context.

In the following sections, we highlight a selection of contributions that illustrate these developments, ranging from coronary and acute care to heart failure, cardiomyopathies, and the wider cardiovascular ecosystem. The overview is not exhaustive; many excellent articles are not discussed in detail. Rather, we aim to capture the main lines of progress visible in the 2025 issues of the *Netherlands Heart Journal* and to reflect on how they may shape clinical practice and research in the years to come.

## Digital health and AI: transforming Dutch cardiology

Several 2025 contributions illustrate how digital health and AI are starting to reshape routine cardiology care in the Netherlands. After hospital discharge for acute coronary syndrome, Vredenburg-Jimmink et al. described a scenario-based, co-designed home-monitoring application that combines education, symptom tracking, and structured follow-up into a digital self-management pathway, demonstrating how post-ACS care can be reorganised around the patient rather than the clinic [1]. In acute heart failure, Bosman et al. reported the DZThuis “Hospital-at-Home” pilot from Deventer Hospital, showing that selected patients receiving intravenous diuretics at home under daily specialist-nurse supervision had mortality, readmission, and complication rates comparable to a historical in-hospital cohort, thereby supporting the feasibility and safety of shifting high-intensity decongestive treatment out of the ward in a hybrid model [2]. At the rhythm level, the HERO registry studied 203 pacemaker recipients with elevated CHA_2_DS_2_-VASc scores and found that more than half experienced device-detected arrhythmias during 5‑year follow-up, quantifying how remote monitoring changes workflows while raising questions about alert burden and resource allocation [3]. A companion commentary by Chiu and Meine explicitly discussed whether remote monitoring in implantable devices is primarily a “care solution” or “alert pollution”, highlighting the importance of carefully designed follow-up pathways when scaling such programmes [4]. Complementing these device-based approaches, Reijrink-de Boer et al. presented initial experience with a virtual atrial fibrillation clinic after pulmonary vein isolation, in which photoplethysmography-based follow-up is used to detect recurrences and organise care largely outside the physical outpatient clinic [5].

Alongside these service innovations, several articles focused on AI-enabled diagnostics and the digital infrastructure around them. Van der Boon and Karper outlined how contemporary echocardiography pipelines are being augmented by machine-learning tools for image selection, view classification, and automated quantification, and discussed the opportunities and caveats of integrating such algorithms into everyday echo labs [6]. From a personal perspective, Van der Harst and Nathoe used the Eko CORE 500 as an example of an AI-supported digital stethoscope that combines high-quality acoustics, ECG recording, and on-device algorithms, illustrating how even the most traditional cardiology symbol is becoming a connected, data-generating sensor [7]. Finally, the editorial introducing nhj.nl framed the launch of a dedicated online companion platform as part of this broader digital transition, positioning the *Netherlands Heart Journal* not only as a venue for studies on telemonitoring and AI, but also as a digital hub for education, implementation tools and rapid dissemination of such innovations [8].

## Optimising coronary and acute care along the continuum

Multiple studies were published on interventional cardiology, reflecting progress in coronary and acute care in the Netherlands. First, three studies were published reflecting contemporary Dutch percutaneous coronary intervention (PCI) care. Camaro (Radboudumc) et al. [9] described the Netherlands Heart Registration (NHR) data on the timing of invasive strategy in patients with non-ST-segment elevation acute myocardial infarction. Invasive catheterization within three days, as endorsed by the Dutch ACS working group [10], is achieved in a very high percentage of patients in both PCI and non-PCI centres. A clearly larger percentage receives PCI within three days when directly admitted to a PCI centre.

Somsen et al. [11] reported the longitudinal (> 10 years) results of a dedicated chronic total occlusion (CTO) PCI program in the Amsterdam UMC, which confirmed that high technical CTO PCI (92%) and procedural success (87%) rates can be achieved by a single center. Renkens et al. described a 5-year outcome subanalysis from the Dutch multicentre, randomised controlled AIDA trial [12]. In these results, lesion-oriented adverse events were low (7.7%) over 5 years, underscoring the efficacy of PCI with contemporary drug-eluting stents.

Outside PCI for coronary artery disease, interventional progress was made in aortic valve stenosis and pulmonary embolism. Schouten et al. described next-day discharge (NDD) after transcatheter aortic valve replacement [13]. An NDD policy was initiated in 2022 in the Antonius Ziekenhuis, and 58.5% of patients were eligible for NDD after TAVR with a very low post-discharge complication rate (1.9% permanent pacemaker implantation and 2.2% minor vascular complications). Hart et al. (UMC Utrecht) reflected on catheter-directed therapy with the FlowTriever system as an alternative for systemic thrombolysis for intermediate high and high-risk pulmonary embolism [14]. Twenty-one patients were treated with the FlowTriever system, and the technical success rate was 100%. Thirty-day all-cause mortality was 29% and major bleeding was recorded in 24%.

## Heart failure and cardiomyopathies in real-world practice

Van der Velde et al. retrospectively evaluated the results of a diagnostic approach, based on the Heart Rhythm Society (HRS) criteria, toward cardiac sarcoidosis. They concluded that 87% of 180 included patients had symptoms, but that the absence of symptoms did not preclude cardiac sarcoidosis. Cardiovascular magnetic resonance imaging (CMR) and positron emission tomography (PET) were performed in 66 and 37% of patients, respectively, cardiac sarcoidosis was confirmed in the minority of patients, and electrocardiography or echocardiography were of limited diagnostic value, although patients without HRS criteria and with normal ECG and echocardiogram had a good prognosis [15]. Subsequently, in a letter to the editor, De Leeuw et al. posed the question of why CMR, as the most accurate diagnostic and prognostic tool, was performed in so few patients [16].

Several studies were presented on the detection and monitoring of heart failure. The incidence of transthyretin amyloid cardiomyopathy, the form of cardiac amyloidosis for which there is effective treatment, was low in an unselected cohort of Dutch all-comer HFpEF patients [17]. Family screening for genetic dilated cardiomyopathy is indicated in the presence of a pathogenic mutation in the index patient. However, among 358 relatives (of whom 41% were (likely) pathogenic variant carriers) DCM was diagnosed in only approximately 10% with a likely pathogenic or pathogenic variant at baseline, and in another 10% of relatives who were re-evaluated during a median follow-up time of 5 years. The authors conclude that screening for DCM in relatives may be limited to variant carriers (“genetic first”), thereby reducing the burden to the healthcare system [18].

The need for early detection of congestion in heart failure was touched upon by Bragança et al., who described the use of non-invasive bioimpedance analysis in 83 ambulatory patients. They showed that approximately 25% of their patients experienced an outcome, most frequently worsening of heart failure. Bioimpedance analysis had a similar predictive power in predicting outcome as NT-proBNP, supporting its use in routine ambulatory heart failure care [19].

Whether or not patients with dilated cardiomyopathy (DCM) and a reduced left ventricular ejection fraction should be implanted with an implantable cardioverter defibrillator (ICD) for primary prevention remains a matter of debate. International guidelines provide a class IIa recommendation for implantation, whereas the Dutch update to the ESC guidelines states that the benefit of primary prevention ICD in most DCM patients is insufficient to justify implantation. In a retrospective analysis on 134 primary prevention ICD patients with non-ischaemic cardiomyopathy, the incidence of appropriate ICD therapy (both antitachycardia pacing and appropriate shocks) was similar in patients deemed to have a low-risk and those without low-risk features (16 versus 12% respectively during a median follow-up of 5.5 years) [20].

The ultimate treatment for end-stage heart failure is heart transplantation. Particularly in patients with a Fontan circulation, heart failure may be accompanied by hepatic failure, which commonly forms a contraindication for heart transplantation. Accord et al. present the clinical course of two patients with a Fontan circulation who were evaluated for combined heart-liver transplantation (CHLT) and conclude that establishing a CHLT program represents a promising therapeutic pathway for patients in the Netherlands with advanced heart failure (HF) and concomitant liver disease [21].

## Dutch cardiovascular ecosystem: consortia, registries, guidelines, and implementation

In 2025 the NHJ published three NVVC endorsements of ESC guidelines, facilitating the integration of international evidence into daily practice while accounting for the Dutch context [22]. In the endorsement paper of the 2023 ESC guideline on cardiovascular disease with diabetes, Martens et al. emphasized proactive screening for type 2 diabetes mellitus (T2DM) in patients with cardiovascular disease (CVD), as well as screening for CVD, chronic kidney disease (CKD), and symptoms of HF in patients with T2DM. Treatment strategies prioritize cardiovascular risk reduction over glycaemic control, with a strong focus on personalized approaches. A stepwise scheme is provided for the prescription of sodium-glucose cotransporter 2 (SGLT2) inhibitors and glucagon-like peptide 1 (GLP1) receptor agonists for risk reduction independent of glucose control, considering Dutch reimbursement criteria [23].

Maass et al. performed a critical appraisal of the 2021 ESC guidelines on cardiac pacing and cardiac resynchronisation therapy in the endorsement paper. In the original guideline, most of the recommendations are class II (61%) and based on expert opinion (59%). An important deviation from the ESC recommendations concerns the cautious approach toward the use of ICD therapy for primary prevention as part of cardiac resynchronization therapy (CRT) in patients with non-ischaemic cardiomyopathy, referring to the previously published “Assessment of ICD eligibility in non-ischaemic cardiomyopathy patients: a position statement by the task force of the Dutch Society of Cardiology” [24, 25]. Two recommendations have been added for implementing His bundle pacing (HBP) and left bundle branch area pacing (LBBAP) based on the 2023 EHRA consensus statement by Burri et al. [26] Harteraad contributed by emphasizing the patient perspective, highlighting that beyond evidence-based recommendations, individual preferences, beliefs, and values should be incorporated to achieve shared decision-making.

The endorsement paper of the 2023 European Society of Cardiology guidelines on the management of cardiomyopathies, by Groeneweg et al., translates ESC recommendations into practical guidance for Dutch cardiologists, tailored to the national healthcare system, shared-care models, and expertise in cardiogenetics and imaging [27]. It introduces key innovations, including refined phenotypic classification and the replacement of imprecise terminology such as “arrhythmogenic cardiomyopathy,” while emphasizing the importance of multidisciplinary care. The paper also provides pragmatic adaptations, such as the selective use of cardiac MRI and genetic testing, to ensure efficient and patient-centred implementation and an overview of relevant recommendations for daily practice in the Netherlands, and advocates a protocolised transition from paediatric to adult cardiology services.

## Sex, gender, and diversity in cardiovascular disease: emerging insights and imperatives

Over recent decades, substantial progress has been made in combating CVD. Yet, significant gaps in our understanding and awareness of CVD in women continue to hinder the adoption of sex- and gender-specific cardiovascular management. Several 2025 publications in the *Netherlands Heart Journal*, spanning the full chain from trial design to daily practice, have shed light on the distinct epidemiological burden, pathobiology, and clinical outcomes of CVD in women. Together, these studies underscore the interplay between sex-specific biological mechanisms and gender-related factors that shape cardiovascular risk and outcomes.

Optimizing cardiovascular health in women demands a sex- and gender-specific, life-course approach to CVD prevention and management. Throughout their reproductive lifespan, women experience profound hormonal and metabolic transitions that influence their cardiovascular risk. Siddiqui et al. re-emphasized the greater impact of several traditional risk factors in women [28]. They also highlighted the underrecognition of female-specific and female-predominant risk-enhancing conditions while assessing cardiovascular risk, including pregnancy-related complications, gynecological disorders, and autoimmune diseases [28].

In another effort to address sex-specific performance of diagnostic algorithms, Li and colleagues evaluated the diagnostic accuracy of the heart failure with preserved ejection fraction (HFpEF) diagnostic algorithms [29]. They included two prospective cohorts with unexplained dyspnoea; the Amsterdam cohort HFpEF confirmed or excluded via (exercise) right heart catheterization (RHC), and the Maastricht cohort HFpEF confirmed or excluded based on expert consensus with utilisation of HFpEF scores, and RHC when needed. Results showed HFpEF diagnostic algorithms to perform reasonably well in ambulatory patients suspected of HFpEF. Compared to males, the algorithms had higher diagnostic performance in females [29].

Prompted by the growing evidence that presentation, progression, and management of atrial arrhythmias, such as atrial fibrillation (AF), differ between women and men, Freriks et al. investigated whether sex differences in the electrophysiological properties of Bachmann’s bundle (BB), assessed by high-resolution and density maps, exist in patients with AF [30]. As BB is the main route for interatrial conduction, the authors hypothesized that sex-related differences in structural and electrical remodeling of BB may contribute to differences in AF development between women and men. They indeed observed a higher proportion of low voltage potentials and more abnormal potential morphologies among women, compared to men [30]. The findings of Freriks and colleagues may reflect sex-specific differences in the underlying substrate of AF at BB.

Advancing sex equity in cardiovascular care hinges on improving the representation of women in clinical trials and expanding research focused on female-predominant cardiovascular conditions. Van der Bijl et al. analysed 115 randomized cardiovascular trials within the WCN investigator network that had cardiovascular events as the primary efficacy endpoint [31]. They showed that women remain structurally underrepresented, with a median participation-to-prevalence ratio below 1 and more than half of the trials under-enrolling women. At the same time, treatment effects on primary endpoints were broadly comparable between women and men [31]. Encouragingly, they concluded that, despite women being underrepresented, their participation in the selected WCN-CVD trials was sufficient to exclude major sex differences in efficacy [31]. Nevertheless, greater female inclusion remains essential for equity and safety insights.

Controversies exist regarding sex differences in outcomes after coronary artery bypass grafting (CABG). Wester ML and colleagues, on behalf of the Cardiothoracic Surgery Registration Committee of the Netherlands Heart Registration, assessed sex differences in early and mid-term outcomes after CABG and factors associated with these differences [32]. Based on data from the Netherlands Heart Registration (NHR), they showed that women undergoing CABG presented with more complex risk profiles, received different surgical strategies, and had worse early and mid-term outcomes compared to men. Female sex was associated with mid-term mortality only in patients < 70 years of age [32]. This illustrates how baseline risk profiles and outcome trajectories differ between women and men even in guideline-driven surgical care.

Collectively, these studies highlight the need for sex- and gender-aware cardiovascular management strategies. To close the sex gap in cardiovascular care, we must ensure meaningful participation of women in clinical trials and prioritize research on the conditions that uniquely or disproportionately affect them. In this line, Kalkman et al. discussed spontaneous coronary artery dissection as an underdiagnosed, predominantly female cause of acute coronary syndrome, summarising its distinct pathophysiology, imaging characteristics, and recurrence risk [33]. Reviewing the national landscape, Dal Canto et al. provided an overview of women’s heart disease research in the Netherlands, with a particular focus on angina with non-obstructive coronary arteries and related phenotypes, positioning ANOCA/INOCA as a key mechanism behind persistent angina in women and mapping the growing Dutch research and funding landscape in this field [34]. They highlighted that the recent Dutch initiatives have established a robust clinical infrastructure and a translational framework that enables us to address some of the gaps in women’s cardiovascular management.

These original studies and reviews are framed by the editorial from Paulis and Kavousi, which explicitly calls for the systematic integration of sex- and gender-specific insights into cardiovascular research, guideline development, and clinical care, and together they define a clear agenda for narrowing the cardiovascular health gap between women and men in the coming years [35]. A national strategy must adopt a comprehensive framework that integrates sex, gender, and broader dimensions of diversity, including ethnicity and socio-economic status. By embracing this inclusive perspective, the goal should be to generate robust evidence that informs tailored strategies and ensures optimal cardiovascular care for all. Consequently, implementation efforts in the Netherlands should prioritize accelerating the integration of sex-, gender-, and diversity-sensitive insights into clinical cardiovascular practice, creating a pivotal opportunity to revolutionize cardiovascular care for everyone.

## Conclusions

The 2025 volume of the *Netherlands Heart Journal* illustrates how Dutch cardiology combines high-quality clinical care with innovation and implementation. Across the themes of sex and gender, digital health and AI, coronary and acute care, and heart failure and cardiomyopathies, the selected papers show a clear shift towards patient-centred, data-driven, and collaborative practice. National and regional registries, DCVA initiatives, and NVVC guideline endorsements provide an infrastructure in which such advances can be tested and scaled.

We are grateful to all authors, reviewers and editors who contributed to the journal in 2025. Building on these foundations, we anticipate that continued attention to sex and gender, responsible use of digital and AI tools, and further strengthening of registry-based and consortium-driven research will help to narrow remaining gaps in cardiovascular outcomes in the years to come.
